# Hereditary thrombocytopenias: the challenge of increasing frequency and differential diagnosis

**DOI:** 10.1016/j.htct.2025.106076

**Published:** 2025-10-29

**Authors:** Rafiye Çiftçiler

**Affiliations:** Selcuk University Faculty of Medicine, Department of Hematology, Konya, Turkey

**Keywords:** Hereditary thrombocytopenia, Acquired thrombocytopenia, Immune thrombocytopenic purpura

## Abstract

Hereditary thrombocytopenias are often difficult to diagnose. Since most of them are rare diseases, their characteristics are less known by physicians who deal with adults. While pediatricians are accustomed to considering genetic diseases in the differential diagnosis of diseases affecting their young patients, clinicians who treat adults often overlook this possibility. Making a definitive diagnosis usually requires complex laboratory techniques. In addition, because this is a very dynamic field half of the patients have forms that have not yet been identified with new disease definitions being made every month with next-generation sequencing (NGS). These patients are often mistakenly diagnosed with immune thrombocytopenia, and, accordingly, they are at risk of receiving unnecessary immunosuppressive therapy. Misdiagnosis is widespread in patients whose low platelet count is discovered in adulthood because, in these cases, the hereditary origin of thrombocytopenia can be overlooked. The age of manifestation and the duration/chronicity of symptoms are crucial clinical features for identifying hereditary thrombocytopenia disorders. It is important to establish the correct diagnosis because it has recently been shown that some hereditary forms of thrombocytopenia predispose to other diseases such as leukemia, renal failure, and bone marrow failure; therefore, affected individuals should be kept under close surveillance and, when necessary, treated for concomitant diseases. This review aims to determine when to suspect and how to diagnose and manage inherited thrombocytopenias. It also intends to detail the less common kinds of isolated thrombocytopenias highlighting that not all isolated thrombocytopenias that emerge in adults are immune thrombocytopenia.

## Introduction

An acquired condition rather than an inherited genetic mutation is more likely to be the underlying cause of newly diagnosed thrombocytopenia in a person of any age. Autoimmune disorders, elevated platelet consumption, splenomegaly, bone marrow suppression (infectious or drug-mediated), and bone marrow failure are among the different reasons for acquired thrombocytopenia [1]. The most frequent causes of acquired thrombocytopenia will differ according to the patient's age at onset, reflecting the prevalent illnesses in each age group. Acute viral infections and ımmune thrombocytopenia (ITP) are more prevalent in children. Because chronic diseases and cancer are more common and diverse in adults, the differential diagnosis is rather extensive [1].

Hereditary thrombocytopenias (HTs) are a rare, heterogeneous group of disorders that result in early-onset thrombocytopenia with marked variability in bleeding. The age at manifestation and the duration/chronicity of symptoms are crucial clinical features for identifying HT disorders. The prenatal period or the time the child starts to crawl and walk are often when acute thrombocytopenia or profound platelet dysfunction is identified. Throughout life, milder abnormalities are observed at periods of hemostatic stress (beginning of menses, surgery/trauma, delivery). However, a significant proportion of individuals with HT may not have clinically significant bleeding and are diagnosed with mild to moderate thrombocytopenia (>20×10^9^/L) during routine blood tests [1]. Because alterations in platelet shape can identify HTs, all patients with recently diagnosed thrombocytopenia should have their peripheral blood smear carefully examined. The diagnostic options are reduced when big platelets or microthrombocytes are identified. Furthermore, due to the fixed particle size, automatic cell counts may underreport very big and very tiny platelets.

In 1948, a patient with Bernard-Soulier syndrome (BSS), a congenital bleeding condition, was described, marking the beginning of the history of HTs [2]. Nearly 70 years later, 33 distinct types of HT caused by genetic abnormalities affecting at least 32 genes were defined as a result of developments in clinical and scientific research [2]. The advent of whole-exome sequencing (WES) and whole-genome sequencing (WGS) significantly advanced our understanding of HTs. This is a very dynamic field with new disease definitions being made every month with next-generation sequencing (NGS). Numerous national and international consortiums devoted to identifying the genes causing HT in patients who did not receive a molecular diagnosis after examining one or more candidate genes have embraced them. These high-throughput sequencing techniques have identified genes that cause illness, such as *PRKACG, GFI1b, STIM1, FYB, SLFN14, ETV6, DIAPH1*, and *SRC* [3, 4, 5, 6, 7, 8, 9, 10, 11]. New genes will continue to be identified in the future. This review aims to determine when to suspect and how to diagnose inherited thrombocytopenias. It also intends to detail the less common kinds of isolated thrombocytopenias highlighting the fact that not all isolated thrombocytopenias that emerge in adults are ITP.

## When to suspect and how to diagnose hereditary thrombocytopenias

Inappropriate therapies and incorrect prognostic criteria might arise from misdiagnosing hereditary illnesses as acquired conditions. Furthermore, misdiagnosis hinders genetic counseling, which might impact subsequent generations. The differential diagnosis between ITP and HT can be challenging due to the absence of specific tests, particularly in patients with mild bleeding symptoms. Recently, the feasibility of using parameters of the complete blood count (CBC) to support this differential diagnosis was illustrated by a series of studies, which demonstrated and validated that the mean platelet volume (MPV) can help the segregation of patients with ITP and HT [12,13]. In recent years, new parameters have been incorporated into the CBC, including the immature platelet fraction (IPF), which represents a population of newly formed platelets containing a greater amount of residual RNA [14]. Initially, the IPF was measured by flow cytometry, and described as reticulated platelets [15]. According to Ferreira et al., IPF helps distinguish hereditary macrothrombocytopenia from ITP; it is elevated in the former when compared to both ITP and other thrombocytopenias [16]. It is still unclear what processes underlie the rise in IPF in hereditary macrothrombocytopenia. One consistent feature linked to the biological causes of thrombocytopenia in these patients may be the higher RNA content of these platelets [16]. Two characteristics of the HTs that are more commonly found in adulthood are that they do not cause spontaneous bleeding and are not syndromic at birth [17]. The abnormalities linked to thrombocytopenia are apparent from the first few months of life in the majority of syndromic types, which makes accurate diagnosis easier [18, 19, 20]. However, in some syndromic types, thrombocytopenia-related problems show up later in life, making it easy to overlook their genetic roots [21,22].

The best diagnostic approach to HTs is still debated. Mutation screening is always necessary for a conclusive diagnosis. Instead of sequencing one or a small number of genes selected based on patient features, a single-step diagnostic process that can assess all potential mutations causing HTs is now feasible. Massive sequencing, however, often identifies some genetic alterations, and it is frequently difficult to discern between pathogenetic and non-pathogenetic variations. With the use of these techniques, new HTs continue to be identified. Because NGS-targeted systems enable the simultaneous investigation of a predetermined set of genes, molecular diagnosis of hereditary illnesses may be completed more quickly and at a lower cost. Ninety percent of patients who had not previously undergone molecular-level investigation were able to have the disease-causing gene detected thanks to a targeted sequencing platform that covered 63 genes associated with thrombotic and bleeding disorders. The platform demonstrated 100 % sensitivity in identifying causal variants previously found by Sanger sequencing [2,23]. An appropriate clinical approach combined with NGS techniques may provide the best chance for diagnostic success. Since NGS is widely used because of its high throughput and high efficiency, additional gene variants linked to HT have been found. It will grow to be a significant addition to first-line diagnostic techniques like the patient’s history, physical examination, and morphological evaluations. Laboratory and clinical features suggestive of HT are shown in Table 1.

**Table 1:** Laboratory and clinical features suggestive of hereditary thrombocytopenia.

**Clinical features suggestive of hereditary thrombocytopenia**
Patients with hereditary thrombocytopenia vary widely in the presence and intensity of their bleeding propensity
Lifelong bleeding/bruising/petechiae symptoms
Presence of thrombocytopenia that is not related to the patient's concomitant disease or medications
Congenital defects with hereditary thrombocytopenias include skeletal deformity, cognitive impairment, central nervous or cardiovascular system malformations, and immunodeficiency
Hereditary thrombocytopenia increases the risk of developing additional diseases, including renal failure or leukemia
Having a family history of bleeding or increased thrombocytopenia
Absence of normal platelet values in a complete blood count
No response or minor response to treatment modalities such as steroids, ıntravenous ımmunoglobulin, anti-D, splenectomy
Response to platelet transfusions shows good growth/normal survival
**Laboratory features suggestive of hereditary thrombocytopenia**
In addition to normal platelet size, small, large, and giant platelets may be seen in peripheral smear
Pale platelets
Döhle bodies, which are protein clumps in neutrophils,
Red cell stomatocytosis
Anemia, high hemoglobin level or high leukocyte count

## Classification of hereditary thrombocytopenias: clinical, biological, and cytogenetic features

**Figure 1:**
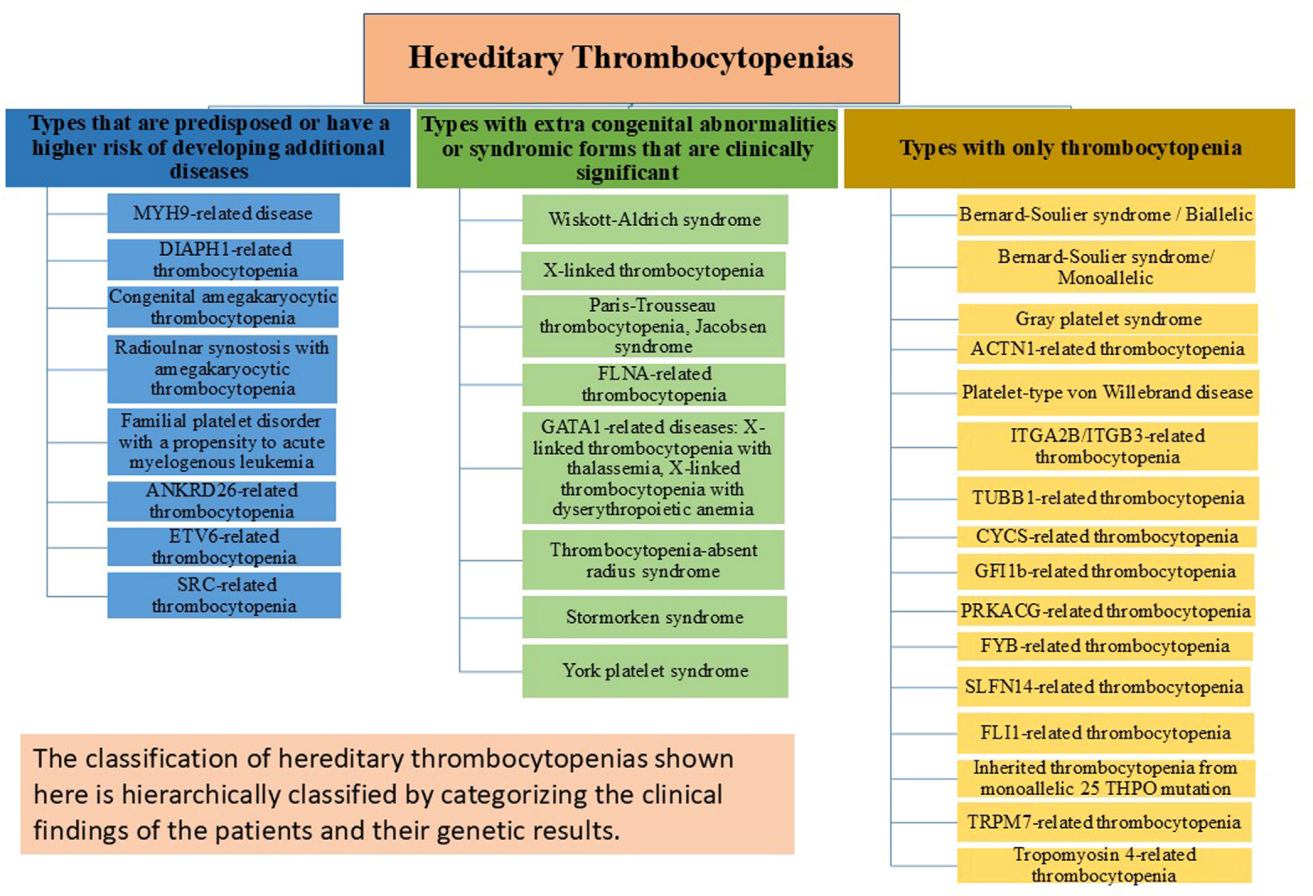
Classification of Hereditary Thrombocytopenias based on more recent perspectives and research.

From minor disorders that may go undiagnosed even in adulthood to severe bleeding diatheses that are identified in the first few weeks of birth, HT has a wide variety of clinical manifestations. To prevent needless and sometimes hazardous therapies, it is crucial to differentiate between HT and acquired thrombocytopenia, particularly ITP, for the category of disorders. An effective diagnosis of HT cannot ignore the meticulous collection of personal and family history, careful physical examination, and analysis of peripheral blood smears. The HT categories that work best for diagnostic reasons are those based on the existence of other abnormalities in addition to thrombocytopenia and platelet size, which vary significantly across the different types. Even if NGS is becoming more widely available, a logical and economical method of diagnosing HTs is still needed. Molecular analysis is necessary to confirm the diagnosis and offer patients individualized treatment, counseling, and follow-up. At the very least, a definite diagnosis must always be sought for persons in whom a predisposing form is suspected. The large number of sporadic instances caused by de novo mutations in some conditions and the inadequate penetrance of the same mutations, frequently making it difficult to discern the inheritance pattern, limits the usefulness of the inheritance-based categorization. In this article, HTs are classified into three groups based on more recent perspectives and research on the condition as depicted in Figure 1: forms that only have the platelet defect; disorders in which the platelet phenotype is associated with other congenital defects (syndromic forms); and forms that have an elevated risk of developing other diseases in life [2,3,11,20,24, 25, 26, 27, 28, 29, 30, 31, 32, 33, 34, 35, 36, 37, 38, 39, 40, 41, 42, 43, 44, 45, 46]. As a result, this categorization has both diagnostic and prognostic value.

### Types that are predisposed or have a higher risk of developing additional diseases

The distribution of hereditary thrombocytopenia types associated with an elevated risk of developing secondary comorbidities or related clinical disorders is delineated in Table 2. A non-syndromic type of macrothrombocytopenia with a tendency to bleed generally correlated with the severity of platelet shortage is the initial manifestation of *myosin-9*-related disease (*MYH9*-RD) [21]. This diagnosis is supported by the finding of Döhle-like structures in the neutrophil cytoplasm [47]. Monoallelic mutations in *DIAPH1*, a gene implicated in proplatelet expansion and cytoskeleton assembly, cause abnormally big platelets in *DIAPH1*-related thrombocytopenia [10]. Congenital megakaryocytic thrombocytopenia (CAMT) typically manifests as isolated, severe, and symptomatic thrombocytopenia from birth. If a hematopoietic stem cell transplant (HSCT) is unsuccessful, this condition almost always progresses into bone marrow aplasia during childhood and results in death before adulthood [44]. In addition to additional skeletal abnormalities and/or sensorineural deafness, radio-ulnar synostosis with amegakaryocytic thrombocytopenia disease is typified by congenital bilateral or unilateral proximal synostosis of the radius and ulna, which results in restricted forearm supination [45]. *ANKRD26*-related thrombocytopenia (*ANKRD26*-RT), *ETV6*-related thrombocytopenia (*ETV6*-RT), and familial platelet disorder predisposing to acute myeloid leukemia (FPD/AML) are three HT linked to specific predispositions to hematological malignancies [13,48]. Platelets in *SRC*-related thrombocytopenia are dysmorphic, exhibit a wide range of sizes, and have few granules [11].

**Table 2:** Hereditary thrombocytopenia types that are predisposed or have a higher risk of developing additional diseases.

Disease name	Genetic transmission type/Gene	The size of platelets	Clinical features
*MYH9*-related disease [24]	AD/*MYH9* (22q12)	Giant, large	Nephropathy, cataracts, and/or sensorineural deafness could develop. Elevated liver enzymes are detected in half of the patients
*DIAPH1*-related thrombocytopenia [10]	AD/*DIAPH1* (5q31.3)	Large	Infantile sensorineural deafness risk. Leukopenia may be moderate and temporary
Congenital amegakaryocytic thrombocytopenia [44]	AR/MPL (1p34.2)	Normal, somewhat diminished	Diminished or nonexistent megakaryocytes. All patients develop significant bone marrow aplasia in infancy
Radioulnar synostosis with amegakaryocytic thrombocytopenia [20,45]	AD/AR**/***HOXA11* (7p15) or *MECOM* (3q26.2)	Normal, somewhat elevated	Megakaryocytes in the bone marrow are diminished or nonexistent. Potential development of bone marrow aplasia. Due to *MECOM* mutations, the hematological phenotype is more severe in the AR variant
Familial platelet disorder with a propensity to acute myelogenous leukemia [46]	AD**/***RUNX1* (21q22)	Normal, somewhat elevated	More than 40 % of patients develop myelodysplastic syndromes or acute myelogenous leukemia. T-cell acute lymphoblastic leukemia risk is elevated. Reduced platelet activity
ANKRD26-related thrombocytopenia[26]	AD/*ANKRD26* (10p12)	Normal, somewhat elevated	Approximately 8 % of patients develop myeloid cancers. Hemoglobin and/or leukocyte counts are elevated in certain cases. Decreased granules in certain patients
ETV6-related thrombocytopenia [9]	AD/*ETV6* (12p13)	Normal, somewhat elevated	Acute lymphoblastic leukemia and other hematological malignancies affect about 25 % of patients
SRC-related thrombocytopenia [11]	AD/*SRC* (20q11.23)	Large	Severe osteoporosis, premature edentulism, juvenile myelofibrosis and splenomegaly, and congenital facial dysmorphism. Agranular or hypogranular platelets. lots of vacuoles

AD: Autosomal dominant; AR: Autosomal recessive.

### Types with extra congenital abnormalities or syndromic forms that are clinically significant

Clinically significant hereditary thrombocytopenia types with extra congenital abnormalities or syndromic forms are listed in Table 3. Almost primarily affecting men, Wiskott-Aldrich syndrome (WAS) is characterized by moderate to severe thrombocytopenia, immunosuppression, and eczema. Microthrombocytes, with a steady platelet count of 5.0–50.0×10^9^/L and a markedly decreased mean platelet volume, are a defining feature of various associated illnesses [49]. In Jacobsen syndrome (and Paris-Trousseau thrombocytopenia, a major and consistent feature of Jacobsen syndrome), probably, the psychomotor retardation and facial and cardiac defects characteristic of Jacobsen syndrome are caused by the loss of a contiguous gene [50,51]. Periventricular nodular heterotopia (PNH) and the otopalatodigital syndrome spectrum diseases, which include congenital deformities, mental retardation, and skeletal abnormalities, are the two primary phenotypes associated with *FLNA* mutations [52]. From birth, patients with *GATA*-related diseases are anemic and have symptoms of severe thrombocytopenia. The bone marrow includes many tiny, dysplastic megakaryocytes in addition to dyserythropoiesis and dysplastic platelet alterations [53]. Bilateral radial aplasia is the defining feature of thrombocytopenia with missing radius syndrome, however, most patients may also have other anatomical abnormalities [54]. The endoplasmic reticulum protein that *STIM1* encodes controls the entry of Ca^2+^ into cells via the calcium release-activated channels of the plasma membrane in a variety of cell types. In Stormorken syndrome and York platelet syndrome, platelets exhibit many in vitro abnormalities related to aggregation, activation, and granule secretion [6,20].

**Table 3:** Hereditary thrombocytopenia types with extra congenital abnormalities or syndromic forms that are clinically significant.

Disease name	Genetic transmission type/Gene	The size of platelets	Clinical features
Wiskott-Aldrich syndrome [37]	XL/WAS (Xp11)	Normal, somewhat diminished	Severe immunodeficiency that results in premature mortality. Eczema. An elevated risk of cancer and autoimmune diseases
X-linked thrombocytopenia [38]	XL/WAS (Xp11)	Normal, somewhat diminished	Mild lack of immunity. mild, temporary eczema. Elevated risk of autoimmunity and cancer. There are descriptions of non-syndromic patients who solely have thrombocytopenia
Paris-Trousseau thrombocytopenia, Jacobsen syndrome [39]	AD**/**Deletions in 11q23	Normal, somewhat elevated	Anomalies of the cardiovascular system, central nervous system, gastrointestinal tract, kidney, and/or urinary tract; growth retardation; cognitive impairment; facial and skull dysmorphisms; and other abnormalities. Reduced platelet activity. decreased dense granules and giant a-granules. Over time, thrombocytopenia may go away.
*FLNA*-related thrombocytopenia [40]	XL/*FLNA* (Xq28)	Large	Heterotopia of periventricular nodules. There are reports of non-syndromic patients who solely have thrombocytopenia.
*GATA1*-related diseases: X-linked thrombocytopenia with thalassemia, X-linked thrombocytopenia with dyserythropoietic anemia [41]	XL/*GATA1* (Xp11)	Normal, somewhat elevated	Hemolytic anemia with splenomegaly, dyserythropoietic anemia, and laboratory abnormalities similar to β-thalassemia. Congenital erythropoietic porphyria.
Thrombocytopenia-absent radius syndrome [42]	AR/*RBM8A* (1q21)	Normal, somewhat diminished	Further anomalies in the upper and lower limbs, as well as bilateral radial aplasia. Potential heart, renal, and/or central nervous system abnormalities. An increase in thrombocytopenia may be linked to a potential cow's milk intolerance. Megakaryocytes in bone marrow are decreased. Over time, the platelet count may increase.
Stormorken syndrome [6]	AD/*STIM1* (11p15)	Not available	Tubular aggregation myopathy, congenital miosis, headache, mild anemia, facial dysmorphisms, anatomical or functional asplenia, ichthyosis, physical growth abnormalities, and cognitive impairment
York platelet syndrome [43]	AD**/***STIM1* (11p15)	Normal	Myopathy, anomalies in the ultrastructure of platelets, including enormous electron-dense and target-like entities, increased vacuoles, and a moderate decrease in granules

AD: Autosomal dominant; AR: Autosomal recessive; XL: X-linked.

### Forms with only thrombocytopenia

Bernard-Soulier syndrome, the most prevalent isolated form of thrombocytopenia, is characterized by recurrent and spontaneous hemorrhages and is frequently fatal since it is linked to severe platelet function abnormalities and thrombocytopenia [28].

Patients with HT vary widely in the presence and intensity of their bleeding tendency. While some patients may only experience hemorrhages during hemostatic challenges, others may present with no bleeding at all. A small percentage of individuals experience spontaneous bleeding at first, and some may even experience potentially fatal bleeding episodes. Petechiae, ecchymoses, epistaxis, menorrhagia, and gastrointestinal hemorrhages are all examples of mucocutaneous bleeding. In those HTs that are not linked to severe platelet dysfunction, the degree of bleeding tendency correlates with the platelet count [24]. On the other hand, in forms linked to altered platelet function, the tendency to hemorrhage is typically more severe than anticipated based on platelet count [28]. For the vast majority of HTs, the severity of thrombocytopenia does not change throughout the patients’ lives. A classification of hereditary thrombocytopenias presenting with isolated thrombocytopenia is provided in Table 4.

**Table 4:** Types of hereditary thrombocytopenia presenting with only thrombocytopenia.

Disease name	Genetic transmission type/Gene	The size of platelets	Clinical features
Bernard-Soulier syndrome/Biallelic [28]	AR/*GP1BA* (17p13) *GPIBB* (22q11)	Giant, large	Platelet function impairment
Bernard-Soulier syndrome/ Monoallelic [25]	AD/*GP9* (3q21)	Large	**–**
Gray platelet syndrome [3]	AR/*NBEAL2* (3p21)	Large	Impaired function of platelets. Greatly decreased granule content. Over time, the platelet count declines. development of splenomegaly and increasing bone marrow fibrosis. Elevated amounts of vitamin B12 in the serum.
*ACTN1*-related thrombocytopenia [29]	AD/*ACTN1* (14q24)	Large	–
Platelet-type von Willebrand disease [30]	AD/*GP1BA* (17p13)	Normal, somewhat elevated	Most patients have a normal platelet count, but in stressful situations (pregnancy, surgery, and infection), it might drop significantly.
ITGA2B/ITGB3-related thrombocytopenia [31]	AD/*TGA2B* (17q21) 1,11/*ITGB3* (17q21)	Large	Impaired function of platelets
TUBB1-related thrombocytopenia [32]	AD/*TUBB1* (20q13)	Large	**–**
*CYCS*-related thrombocytopenia [33]	AD/*CYCS* (7p15)	Normal, somewhat diminished	**–**
*GFI1b*-related thrombocytopenia [5]	AD/*GFI1B* (9q34)	Large	Impaired function of platelets. Granules are reduced. Anisocytosis of red cells
*PRKACG*-related thrombocytopenia [4]	AR/*PRKACG* (9q21)	Large	Impaired function of platelets
*FYB*-related thrombocytopenia [7]	AR/*FYB* (5p13.1)	Normal, somewhat diminished	**–**
*SLFN14*-related thrombocytopenia [8]	AD/*SLFN14* (17q12)	Normal, large	Impaired function of platelets
*FLI1*-related thrombocytopenia [34]	AR/*FLI1* (11p24.3)	Large	Impaired function of platelets. Enormous granules.
Inherited thrombocytopenia from monoallelic 25 *THPO* mutation [35]	AD/*THPO* (3q27.1)	Normal, somewhat elevated	**–**
*TRPM7*-related thrombocytopenia [36]	AD/*TRPM7* (15q21.2)	Large	Increasing number of microtubules, anarchic microtubule architecture, and aberrant granule dispersion.
Tropomyosin 4-related thrombocytopenia [27]	AD/*TPM4* (19p13.1)	Large	**–**

AD: Autosomal dominant; AR: Autosomal recessive

## Treatment and follow-up of individuals with hereditary Thrombocytopenias

The treatment and follow-up of these patients is a very important issue in itself and should be discussed in detail in another article. Avoiding the use of medications that might reveal the platelet abnormality is a crucial first step [55]. Thus, it is important to carefully weigh the advantages and disadvantages of various therapies. Preventing the need for medical procedures that increase the risk of bleeding is another crucial step. New therapy avenues in this area were made possible by the recent discovery that eltrombopag, an oral nonpeptide agonist of the thrombopoietin receptor, might raise the platelet count in a small case series of individuals with *MYH9*-RD [56]. Patients with HTs who are predisposed or have a higher risk of developing additional diseases need close follow-up, whereas individuals with HTs who are not at risk of developing new disorders need medical care for hemostatic problems or bleeding episodes [57].

## Conclusions and future perspectives for the hereditary thrombocytopenias

Once thought to be rare, HT syndromes are now more widely recognized as a group of clinical abnormalities ranging from mild disorders discovered incidentally in adults to severe diseases in neonates. In recent years, our knowledge of HTs has shown that these syndromes will not only present with thrombocytopenia and bleeding disorders, but also with complex diseases and even malignancies, bone marrow failure, and renal, hearing, and vision disorders. A multidisciplinary approach to patients with HT is needed, taking into account additional disorders that have already developed or are at risk of developing soon. The classification of HTs should be evaluated together with the clinical findings of the patient and categorized together with the genetic results. Studying the clinical features of patients and identifying new genes and their association will greatly contribute to the understanding of the pathophysiology of HT syndromes and the development of new treatments.

## Funding information

No funding was received for this article.

## Conflicts of interest

No competing financial interests exist.
